# Fundus Autofluorescence Imaging in Patients with Choroidal Melanoma

**DOI:** 10.3390/cancers14071809

**Published:** 2022-04-02

**Authors:** Almut Bindewald-Wittich, Frank G. Holz, Thomas Ach, Miltiadis Fiorentzis, Nikolaos E. Bechrakis, Gregor D. Willerding

**Affiliations:** 1 Augenkompetenz Zentren Heidenheim, 89518 Heidenheim, Germany; 2 Augenkompetenz Zentren Bopfingen, 73441 Bopfingen, Germany; 3 Department of Ophthalmology, University of Bonn, 53127 Bonn, Germany; frank.holz@ukbonn.de (F.G.H.); thomas.ach@ukbonn.de (T.A.); 4 Department of Ophthalmology, University Hospital Essen, University of Duisburg-Essen, 45147 Essen, Germany; miltiadis.fiorentzis@uk-essen.de (M.F.); nikolaos.bechrakis@uk-essen.de (N.E.B.); 5 Department of Ophthalmology, DRK Kliniken Berlin Westend, 14050 Berlin, Germany; g.willerding@drk-kliniken-berlin.de

**Keywords:** choroidal melanoma, choroidal nevus, brachytherapy, fundus autofluorescence, lipofuscin, melanolipofuscin, retinal pigment epithelium, scanning laser ophthalmoscopy, retina

## Abstract

**Simple Summary:**

The ocular fundus contains molecules that emit fluorescence when excited with light of an appropriate wavelength. Fundus autofluorescence imaging is based on the in vivo detection of intrinsic fluorescence and results in topographic autofluorescence mapping of the ocular fundus. In contrast to fluorescence angiography, where the fluorescing agents need to be administered intravenously, autofluorescence imaging is a non-invasive technique. Even though choroidal melanomas do not contain significant autofluorescent molecules themselves, they may lead to secondary alterations in neighbouring tissues with an impact on the autofluorescence signal recording. Fundus autofluorescence imaging in the context of choroidal melanoma is helpful for differential diagnosis and for monitoring variations over time in affected patients before and after treatment.

**Abstract:**

Choroidal melanocytic lesions require reliable and precise clinical examination and diagnosis to differentiate benign choroidal nevi from choroidal melanoma, as the latter may become life-threatening through metastatic disease. To come to an accurate diagnosis, as well as for monitoring, and to assess the efficacy of therapy, various imaging modalities may be used, one of which is non-invasive fundus autofluorescence (FAF) imaging using novel high-resolution digital imaging technology. FAF imaging is based on the visualization of intrinsic fluorophores in the ocular fundus. Lipofuscin and melanolipofuscin within the postmitotic retinal pigment epithelium (RPE) cells represent the major fluorophores that contribute to the FAF signal. In addition, the presence or loss of absorbing molecular constituents may have an impact on the FAF signal. A choroidal melanoma can cause secondary retinal and RPE alterations that affect the FAF signal (e.g., occurrence of orange pigment). Therefore, FAF imaging supports multimodal imaging and gives additional information over and above conventional imaging modalities regarding retinal metabolism and RPE health status. This article summarises the features of FAF imaging and the role of FAF imaging in the context of choroidal melanoma, both before and following therapeutic intervention.

## 1. Introduction

Choroidal melanoma is a potentially metastasising, life- and sight-threatening disease [1,2]. Therefore, early diagnosis, monitoring of tumour growth, and follow-up after therapy are indispensable to vision and life. Today, in clinical routine, a broad spectrum of imaging technologies is applied for the best possible care of patients with choroidal melanoma [3,4,5,6]. Multimodal imaging includes fundus photography, infrared imaging, fundus autofluorescence (FAF) imaging, fluorescein and indocyanine green angiography, ultrasonography, and optical coherence tomography. This article reviews the role of digital FAF imaging in patients with choroidal melanoma and reflects the importance of FAF imaging in both the differential diagnosis and the management of choroidal melanoma.

## 2. Fundus Autofluorescence Imaging

Non-invasive in vivo FAF imaging is based on the visualization of endogenous fluorophores in the ocular fundus [7,8,9]. The first scanning laser ophthalmoscope was presented in 1980 by Webb et al. and continuously improved in the following years [10,11,12]. FAF imaging using confocal scanning laser ophthalmoscopy (cSLO) was described in 1995 [13,14,15]. Since then, continuous and further developments have strengthened FAF imaging to become a safe and reproducible imaging method that is essential for the routine clinical examination of various chorioretinal diseases [16]. Nowadays, different commercially available devices are used to record FAF [17]. They may not be absolutely equivalent and comparable. Therefore, it is essential to understand the basic principles of FAF imaging techniques and to know the different imaging systems.

### 2.1. Basic Principles

The term fundus autofluorescence describes the natural transient emission of light by the ocular fundus following excitation by light. More detailed, specific molecules absorb energy and achieve a higher energy level when excited by light of a certain wavelength. These excited molecules then relax to a lower energy state through the emission of light (photons) with a longer wavelength than the excitation light. The different wavelength ranges of the excitation light and the emitted light allow for the detection of the fluorescence signal using appropriate filters.

### 2.2. Retinal Pigment Epithelium and Main Fluorophores

In the human eye, the main fluorophores are multiple constituents of intracellular lipofuscin and melanolipofuscin granules at the level of the postmitotic retinal pigment epithelium (RPE) [18,19,20,21,22,23]. The RPE is a monolayer of polygonal-shaped, mitotically quiescent cells between the photoreceptor layer and Bruch’s membrane/choriocapillaris complex [24,25]. It possesses various essential physiological functions, such as the absorption of light; the transport of water, ions, and metabolites from the subretinal space to the blood; the transport of nutrients from choroidal circulation to the outer retina, including light-sensitive photoreceptors; and the secretion of growth factors [26]. The lifelong phagocytosis of debris from the daily shedding of photoreceptor outer segments by the RPE [27,28] and the re-isomerization from all-*trans*-retinol to 11-*cis*-retinal as part of the visual cycle play an important role in the lipofuscinogenesis [29,30,31]. Hence, the major fluorophores are by-products of the visual cycle. They consist of a mixture of bisretinoid molecules and accumulate as non-degradable lipofuscin and melanolipofuscin granules in the lysosomal compartment of the RPE with age and in association with various chorioretinal diseases [32,33].

### 2.3. Imaging Devices

The FAF signal of a normal ocular fundus is weak. Autofluorescence from other tissues, as well as the absorption of excitation and emitted light, especially by the crystalline lens, may confound the detection of FAF. Therefore, appropriate imaging technology is required to amplify the FAF signal and to record images of adequate quality and contrast [16,34]. Different devices need to be considered, which differ in their applied excitation and emission wavelengths, scanning mode, and image acquisition.

#### 2.3.1. Scanning Laser Ophthalmoscopy

Confocal scanning laser ophthalmoscopy (cSLO) uses a continuous illumination of the ocular fundus with a low-power, blue-light laser beam that scans the fundus in a raster pattern (excitation wavelength 488 nm, 490 nm, or 450 nm, depending on the manufacturer). An appropriate barrier filter (cut-off filter) ahead of the detector ensures the blockade of reflected excitation light and the detection of the emitted light (e.g., SPECTRALIS HRA2, Heidelberg Engineering, Heidelberg, Germany: >500 nm; F-10 and Mirante Systems, NIDEK, Gamagori, Japan: 510 nm for blue short-wave FAF, 630 nm for green short-wave FAF; EIDON, Centervue, Padova, Italy: 510–560 nm and 560–700 nm). The confocal principle is based on a small pinhole in front of the detector. Thus, back-scattered light originating from structures anterior and posterior to the focal plane is suppressed, and emitted light from the desired plane of focus can be amplified to enhance image contrast [35]. The maximal retinal irradiation is well below the ANSI Z136.1 laser safety standard [36].

#### 2.3.2. Fundus Camera

A modified fundus camera was used to detect FAF by Delori and co-workers [37]. Later, a modification of a commercially available fundus camera system was applied to FAF imaging [38]. In contrast to confocal scanning laser ophthalmoscopy, non-confocal fundus camera systems use one high-energy flash at maximum intensity. For excitation and the detection of emission, a wide-band filter is used (e.g., TRC-50DX/50IX, Topcon, Tokyo, Japan: excitation wavelength range 500–610 nm, detection of emission 675–715 nm; Visucam 224/524/FF450, Carl Zeiss Meditec AG, Jena, Germany: excitation wavelength range 510–580 nm, detection of emission 650–735 nm; CX-1/CR-2, Canon, Tokyo, Japan: excitation wavelength range 530–580 nm, detection of emission >640 nm).

More recent systems use broad line fundus imaging (BLFI) technology with illumination in two broad wavelength ranges (FAF-Blue: excitation 435–500 nm, FAF-Green: excitation 500–585 nm) and the detection of emission within a band-pass filter range of 532–650 nm (for FAF-Blue) or 630–750 nm (for FAF-Green) (ZEISS CLARUS 500/700, Carl Zeiss Meditec AG, Jena, Germany).

#### 2.3.3. Wide-Field Imaging

As choroidal melanoma can occur on all sides of the ocular fundus, visualization of the central and peripheral retina is of importance. In conventional scanning laser ophthalmoscopy systems, the field of view is restricted to 60° (EIDON), 40° or 60° (F-10/Mirante), or 30° (SPECTRALIS HRA2). The latter may be extended to 55° using a wide-field lens. Composite images may be able to image larger areas.

The fields of view of different fundus camera systems vary between 20° and 50°. Montage images of larger areas can be manually generated. The ZEISS CLARUS 500 and 700 achieve a 133-degree field of view with the possibility of 267-degree montage images. For peripheral FAF images, another non-mydriatic, non-confocal ultra-wide-field scanning laser ophthalmoscope provides up to approximately 180–200° images of the retina in a single capture using a green laser for excitation (532 nm) and a detection of emission in the range of 540–800 nm (Optomap, Optos PLC, Dunfermline, UK) [39]. Ultra-wide-field imaging is of particular importance to visualize lesions anterior to the equator [40,41,42].

### 2.4. Interpreting Fundus Autofluorescence Images

The grey value of each pixel depends on the intensity of the FAF signal, with low intensities resulting in low pixel values, which appear dark, and high intensities resulting in high pixel values, which appear bright. A reduced FAF signal arises from absorption effects or from the loss or absence of fluorophores (e.g., RPE atrophy). Absorption phenomena occur in the area of retinal vessels, due to luteal pigment, i.e., lutein and zeaxanthin, an increased RPE melanin content, and migrated melanin-containing cells. Absorption may further be caused by non-fluorescent extracellular material anterior to the RPE (e.g., recent fluid or haemorrhage, media opacities, lens opacifications). In contrast, an increased FAF signal may derive from augmented RPE lipofuscin/melanolipofuscin accumulation, deposited fluorophores (e.g., vitelliform material, orange pigment), a lack of absorbing material, a loss of photopigment, lipofuscin-containing macrophages, devitalized haemorrhages [43], or RPE cell migration. Long-lasting subretinal fluid is associated with a malfunctioning outer segment turnover between photoreceptors and the RPE and can also cause an increased FAF signal [7,16,44,45].

## 3. Choroidal Melanoma

### 3.1. General Aspects

Choroidal melanoma is the most common primary intraocular malignancy emanating from choroidal melanocytes. The incidence is approximately 5-6 new cases per million per year, depending on ethnicity and age, with a median age of 55 years [46,47,48]. Metastases most often affect the liver, followed by the lungs, bone, and skin. In 8033 consecutive eyes, the metastatic risk at 10 years has been indicated at 12% for small melanoma (<3-mm thickness), 26% for medium-sized melanoma (3.1- to 8-mm thickness), and 49% for large melanoma (>8-mm thickness) [49]. Given a mortality rate of approximately 50% over the next 10 years in patients with large choroidal melanoma, early detection is crucial [50,51]. Clinically, choroidal melanomas present as pigmented, non-pigmented, or mixed dome-shaped lesions. They may display a mushroom-like appearance when breaking through Bruch’s membrane. Today, the diagnosis of choroidal melanoma is mainly based on clinical examination supported by multimodal imaging. Invasive fine-needle biopsy, vitrectomy biopsy, or incisional/excisional biopsy for histologic or cytologic evaluation are not routinely performed due to the risk of intraocular complications, extraocular dissemination, and a low sensitivity rate [52]. The potential of blood liquid biopsies for choroidal melanoma is still under evaluation [53].

### 3.2. To Differentiate between Choroidal Melanoma and Choroidal Nevus

**Figure 1:**
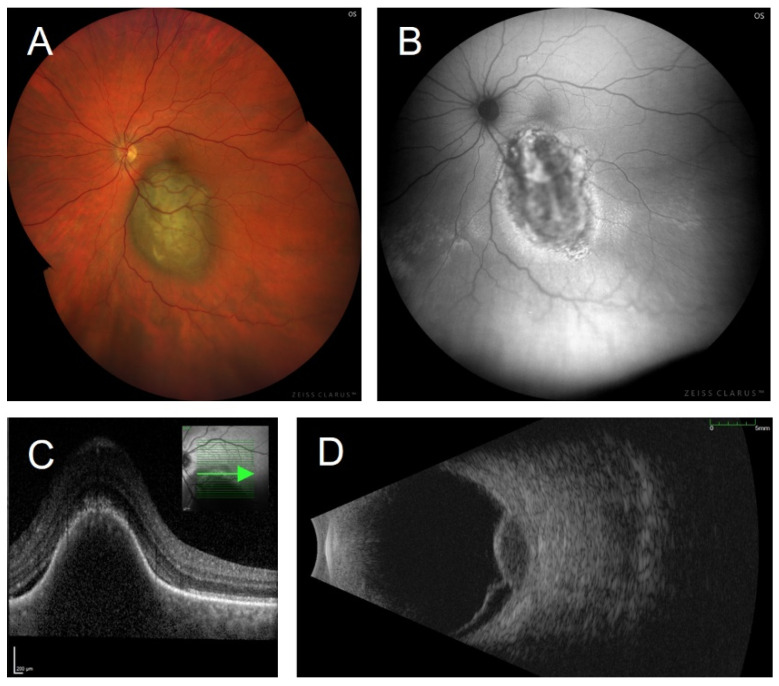
Multimodal imaging of a choroidal melanoma in a 51-year-old male patient presenting with metamorphopsia. Visual acuity was 20/20. (**A**) The fundus photograph shows a pigmented lesion (diameter > 5 mm) with overlying orange pigment, and (**B**) with marked changes of the signal seen on fundus autofluorescence (illumination with 500–585 nm, FAF-Green). Areas of intensely increased fundus autofluorescence alternate with areas of decreased fundus autofluorescence, reflecting a pronounced retinal pigment epitheliopathy, the presence of orange pigment, and subretinal fluid. (**C**) An OCT scan at the tumour’s margin shows a subretinal cleft due to malignant exudation. (**D**) Exudation is also seen on ultrasonography as exudative retinal detachment. The prominent tumour (height 3.6 mm) exhibits acoustic hollowness as a sonographic hallmark of choroidal melanomas. Images were taken using (**A**,**B**) the ZEISS CLARUS 700 (Carl Zeiss Meditec AG, Jena, Germany), (**C**) the SPECTRALIS OCT (Heidelberg Engineering, Heidelberg, Germany), and (**D**) Eye Cubed ^TM^ (Ellex Inc., Minneapolis, MI, USA).

Differential diagnosis between small choroidal melanoma and choroidal nevi in clinical examination can be challenging. Various quantitative factors (largest basal diameter, tumour height) and qualitative prognostic factors (subretinal fluid, RPE changes, orange pigment, drusen) have been considered to establish objective criteria for the early identification of choroidal melanoma. The latest key features are based on a retrospective chart review and follow-up of 2355 cases [54]. A multivariate analysis was performed with respect to multimodal imaging features. Shields et al. identified the following risk factors for the transformation of choroidal nevi into melanoma: tumour thickness > 2 mm (ultrasonography), the presence of subretinal fluid (optical coherence tomography, OCT), symptomatic vision loss (Snellen visual acuity), an orange pigment overlying the tumour (FAF imaging), acoustic hollowness (ultrasonography), tumour diameter > 5 mm (photography). These criteria can be remembered with the mnemonic, “To Find Small Ocular Melanoma Doing IMaging” (TFSOM-DIM), representing Thickness > 2 mm, subretinal Fluid, Symptoms (vision loss), Orange pigment, Melanoma hollow, and DIaMeter > 5 mm (Figure 1). An increasing number of these risk factors was associated with a higher risk for transformation. The authors report a mean 5-year estimate of nevus transformation into melanoma of 1% for those with zero risk factors, 11% with one risk factor, 22% with two factors, 34% with three factors, 51% with four factors, 55% with five factors, and not estimable for those with six risk factors [54]. The former features (tumour margin ≤ 3 mm to the optic disc, absence of overlying drusen, and absence of a surrounding halo [55,56,57,58]) were not significant in this multivariate analysis. However, in contrast to formerly exclusive ophthalmoscopy, the establishment of six risk factors benefits from multimodal imaging, including the detection of orange pigment by means of FAF imaging.

### 3.3. Tumour-Associated Retinal Pigment Epitheliopathy

It has been questioned whether choroidal melanoma cells contain autofluorescing molecules. Microscopic examinations performed by Lohmann and colleagues revealed only low endogenous fluorescence intensities of choroidal melanomas (excitation light 365 nm, emission between 340 and 380 nm) [59]. Using fluorescence microscopy, Gündüz and co-workers did not find autofluorescence within a choroidal melanoma [60]. Therefore, FAF imaging is not predestined to image the tumour itself underneath the RPE. However, as shown by microscopic studies, there are secondary changes within the RPE cell monolayer overlying choroidal melanomas [61,62,63,64]. Especially small and active melanomas present with an overlying orange pigment, whereas with time and tumour growth, the RPE degenerates in terms of hyperplasia, atrophy, and fibrous metaplasia [58,65]. Furthermore, RPE cells overlying choroidal melanomas show reactive changes that lead to morphological transformation and the formation of a multi-layered RPE [64]. Clinically and histologically significant abnormalities of the RPE in the presence of choroidal tumours, including RPE proliferation, detachment, atrophy, metaplasia, and accumulation of lipofuscin/melanin [62], have also been termed “tumour-associated retinal pigment epitheliopathy”. These secondary retinal and RPE changes are responsible for subsequent alterations of the FAF signal. Hence, FAF imaging gives additional information regarding the health and metabolic status of the overlying RPE and the retina in the presence of choroidal melanoma. This is of special importance in the case of a small or newly diagnosed choroidal melanoma to assess tumour activity [66].

### 3.4. Orange Pigment

**Figure 2:**
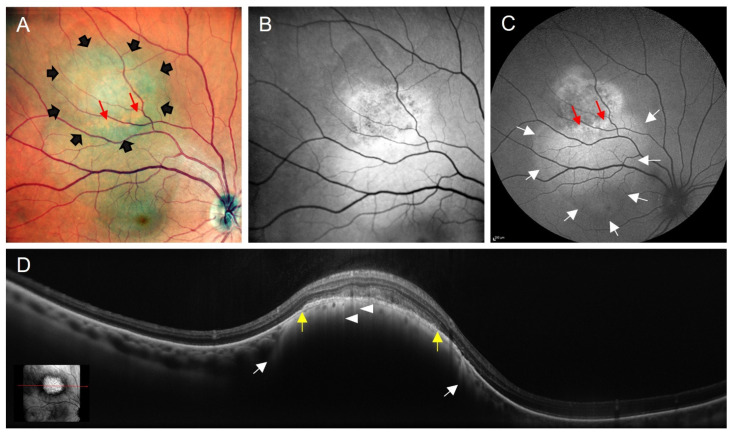
Multimodal imaging of a choroidal melanoma in a 58-year-old Caucasian male. (**A**) Fundus photography. Composite of three separate red/green/blue detectors simultaneously scanning different depths of the retina with red, green, and blue wavelengths, showing choroidal melanoma in the midperiphery (delineated by black arrowheads). Orange pigment (red arrows) can clearly be seen on top of the tumour mass. (**B**,**C**) Fundus autofluorescence images from different devices, both using 488 nm for excitation. The tumour has areas with increased and decreased autofluorescence. Orange pigment (red arrows) presents with brightly increased autofluorescence. Subretinal fluid—as an additional clinical sign of malignancy—has led to an area with increased autofluorescence (delineated by white arrows) adjacent to the tumour and involving the fovea. (**D**) SD-OCT scan through the choroidal melanoma (see inlet for exact location of the scan) shows the prominent tumour mass with compression of the choriocapillaris (between the two white arrows). In addition, the retinal pigment epithelium overlying the tumour (between the yellow arrowheads) is elevated from Bruch’s membrane. Tiny areas of RPE layer thinning or atrophy led to hypertransmission of the OCT signal (white arrowheads). Images were taken using: (**A**,**B**,**D**) the Mirante SLO/OCT (Nidek, Gamagori, Japan) and (**C**) the SPECTRALIS HRA (Heidelberg Engineering, Heidelberg, Germany) platforms.

Decades ago, orange pigment or “lipofuscin pigment” was described in association with both benign and malignant choroidal tumours and was hypothesised to accumulate in RPE cells and macrophages [67,68,69]. However, the precise cellular and subcellular localisation was in question. Immunohistochemistry with the macrophage marker CD68 may be misleading because CD68 can be found on the surface of RPE cells [70,71]. Another study combined fluorescence microscopy, immunohistochemistry, and electron microscopy. Using a CD163 stain, Garcia et al. could confirm that macrophages were sparse in areas with lipofuscin accumulation. Macrophages occurred in the fluid of retinal detachment, whereas orange pigment was correlated with proliferating, clustered RPE cells with intracellular lipofuscin and melanolipofuscin granules [64]. Depending on the pigmentation, orange pigment clinically presents as focal, yellow-orange to red-brown debris on the surface of intraocular tumours, but may be discreet and almost invisible [58]. Orange pigment is not pathognomonic for choroidal melanoma, but can occasionally occur overlying other choroidal pathologies, such as choroidal nevi, carcinoma metastases, and choroidal haemangiomas [67,69,72]. Detected with ophthalmoscopy and colour fundus photography, it has long been postulated to be a risk factor for the growth of choroidal melanocytic lesions [55,73,74,75]. Later, orange pigment has been shown to possess autofluorescent properties [58,66,76,77,78]. It is associated with a mild to marked increase in the FAF signal (Figure 2). As mentioned above, the presence of orange pigment is one of six prognostic factors associated with the malignant transformation of choroidal melanocytic lesions into choroidal melanoma [54]. Compared to fundus photography, FAF imaging is capable of visualizing subclinical orange pigment, which may be important for the early detection of choroidal melanoma [66,76,79].

Increased FAF on the tumour’s surface is not automatically equivalent with orange pigment, but may also derive from pigmentation, fibrous metaplasia, as well as sub-RPE drusen [65], and may furthermore interfere with subretinal fluid, intraretinal oedema, the presence of RPE detachment, and the loss of absorbing factors. Hence, all sorts of FAF findings require precise and experienced interpretation.

### 3.5. Fundus Autofluorescence Imaging of Choroidal Melanoma

When using FAF imaging in the presence of choroidal melanoma, the first striking aspect has been the detection of brightly fluorescing orange pigment. Shields et al. reported the FAF of orange pigment in four patients with small choroidal melanoma in 2007 using a fundus camera with an excitation wavelength of 580 nm and a detection of emission > 695 nm [66]. Gündüz et al. overlaid clinically visible orange pigment (*n* = 13), hyperpigmentation (*n* = 9), drusen (*n* = 6), and fibrous metaplasia (*n* = 4) on colour fundus photographs with areas of increased FAF on FAF-mean images in a total of 23 patients [76]. The best correlation for orange pigment with increased FAF (61.5%) was found using a confocal scanning laser ophthalmoscope (excitation wavelength 488 nm, detection of emitted light > 500 nm; HRA, Heidelberg Engineering, Heidelberg, Germany). The authors conclude that FAF imaging may be more sensitive for the detection of subtle orange pigment than clinical examination. In another report, two different FAF patterns have been described overlying choroidal melanocytic lesions using the same technical device [80]. The patchy FAF pattern consists of areas of increased FAF between areas of normal FAF and was associated with choroidal nevi. In contrast, the diffuse FAF pattern, characterised by increased FAF with indistinct borders, was significantly associated with choroidal melanoma, increased tumour thickness, and increased tumour base diameter. The authors of this study state that the FAF pattern may be important for the clinical differentiation between choroidal nevi and choroidal melanoma and may have an impact on the decision to treat [80]. This study holds some limitations regarding the small study population (choroidal nevi *n* = 11, choroidal melanomas *n* = 19) and the grading of FAF images by two different observers, which was not performed in a masked fashion regarding the identity of the patients.

The patchy and diffuse FAF patterns were demonstrated in another study with FAF imaging in the presence of intraocular tumours using an excitation band-pass filter (535–585 nm) and a barrier band-pass filter for the detection of emission (605–715 nm) (TRC-50DX, Topcon, Tokyo, Japan) [81]. In choroidal melanoma, both the patchy (*n* = 6) and the diffuse (*n* = 4) FAF pattern was found. Significant differences between choroidal nevi (*n* = 19) and choroidal melanomas (*n* = 10) regarding theses FAF findings were found, assuming that choroidal nevi present with less chronic RPE degenerative features than choroidal melanomas. Again, there was only a small number of patients included with choroidal melanoma.

Using confocal scanning laser ophthalmoscopy (HRA2, Heidelberg Engineering, Heidelberg, Germany), Lavinsky et al. described the FAF features of choroidal nevus (*n* = 15) and choroidal melanoma (*n* = 5). Most nevi were associated with normal FAF, whereas choroidal melanomas showed confluent to plaque-like increased FAF due to orange pigment [65]. Cennamo et al. described FAF changes associated with choroidal nevi (*n* = 100) as normal (*n* = 40) or decreased (*n* = 60), whereas choroidal melanomas (*n* = 65) showed plaque-like increased FAF and surrounding decreased FAF in 26 eyes (SPECTRALIS HRA+OCT, Heidelberg Engineering, Heidelberg, Germany) [79].

Shields et al. reported FAF characteristics overlying choroidal nevi (*n* = 64) and choroidal melanomas (*n* = 51) using fundus-camera-based autofluorescence photography (excitation wavelength 580 nm, detection of emission > 695 nm) [82,83]. Choroidal nevi appeared with decreased FAF in 56%, normal FAF in 19%, and increased FAF in 25%, whereas choroidal melanomas showed decreased FAF in 39%, normal FAF in 6%, and increased FAF in 55%. Decreased FAF was associated with pigmented nevi, whereas nonpigmented nevi showed slightly increased FAF due to the unmasking of deeper scleral FAF.

Using non-mydriatic, non-confocal wide-field retinal imaging (green excitation wavelength 532 nm) (Optomap Panoramic 200 Tx, Optos PLC, Dunfermline, UK), Reznicek et al. measured FAF intensities using the software Image J [84]. The mean FAF intensity of choroidal melanomas was significantly lower than the mean FAF intensity of choroidal nevi, and FAF irregularity was lower in eyes with choroidal melanoma versus in eyes with choroidal nevus. Due to a low sensitivity, FAF intensity alone was not adequate to differentiate choroidal melanoma from choroidal nevus. The main characteristics of the above-mentioned studies are summarised in Table 1.

**Figure 3:**
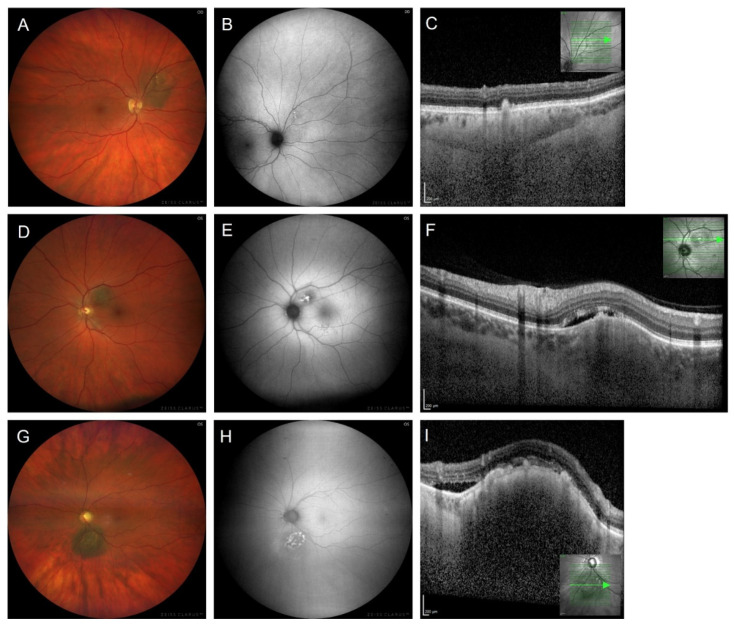
(**A**–**C**) Imaging of a choroidal nevus and (**D**–**I**) of small choroidal melanomas. (**A**) Fundus photograph and (**B**) fundus autofluorescence image of a choroidal nevus in a 61-year-old female. Only mild changes of the normal fundus autofluorescence signal occur. (**C**) A corresponding SD-OCT scan shows drusen but does not reveal any subretinal fluid. Prominence in ultrasound measurements (not shown) was <1 mm. (**D**–**F**) Small choroidal melanoma in a 56-year-old male patient with tumour height being <1 mm (ultrasonography not shown). (**G**–**I**) Small choroidal melanoma in a 56-year-old male with a tumour height of 1.35 mm (ultrasonography not shown). (**E**,**H**) Fundus autofluorescence shows a markedly increased signal due to orange pigment concomitant with areas of decreased autofluorescence overlying the choroidal melanoma. (**F**,**I**) On corresponding SD-OCT scans, subretinal fluid and retinal pigment epitheliopathy are found. Images were taken using (**A**,**B**,**D**,**E**,**G**,**H**) the ZEISS CLARUS 700 (Carl Zeiss Meditec AG, Jena, Germany) and (**C**,**F**,**I**) the SPECTRALIS OCT (Heidelberg Engineering, Heidelberg, Germany). (**B**,**E**,**H**): illumination with 500–585 nm (FAF-Green).

Summing up, the differential diagnosis between choroidal nevus and choroidal melanoma is important. Reports on FAF changes in association with choroidal nevus remain somehow controversial. Differences may be due to the varying pigmentation, variable activity, and chronicity of choroidal nevi, e.g., overlying RPE alterations in chronic stable lesions versus active nevi with new-onset subretinal fluid being suggestive of a transformation into melanoma. Some authors, therefore, differentiate suspicious nevi or indeterminate choroidal melanocytic lesions [81,85]. There is consensus that choroidal nevi rarely show brightly increased FAF due to orange pigment, whereas remarkably increased FAF (ranging from mildly to intensely) overlying choroidal melanomas derives from orange pigment, and less bright FAF from persistent subretinal fluid, drusen, RPE hyperplasia, fibrous metaplasia, and old haemorrhage (Figure 3) [58,77]. In contrast, a decreased FAF signal can be caused by RPE atrophy overlying the tumour, recent haemorrhage, or the absorbing effects of recent subretinal fluid [60,77]. Additionally, in the presence of nonpigmented melanomas, a lack of pigment may unmask scleral autofluorescence, whereas an increased pigment content blocks scleral autofluorescence [58]. There is agreement that drusen overlying choroidal melanocytic lesions may be associated with variable FAF changes, such as drusen in age-related macular degeneration [86].

### 3.6. Fundus Autofluorescence Imaging following Treatment of Choroidal Melanoma

**Figure 4:**
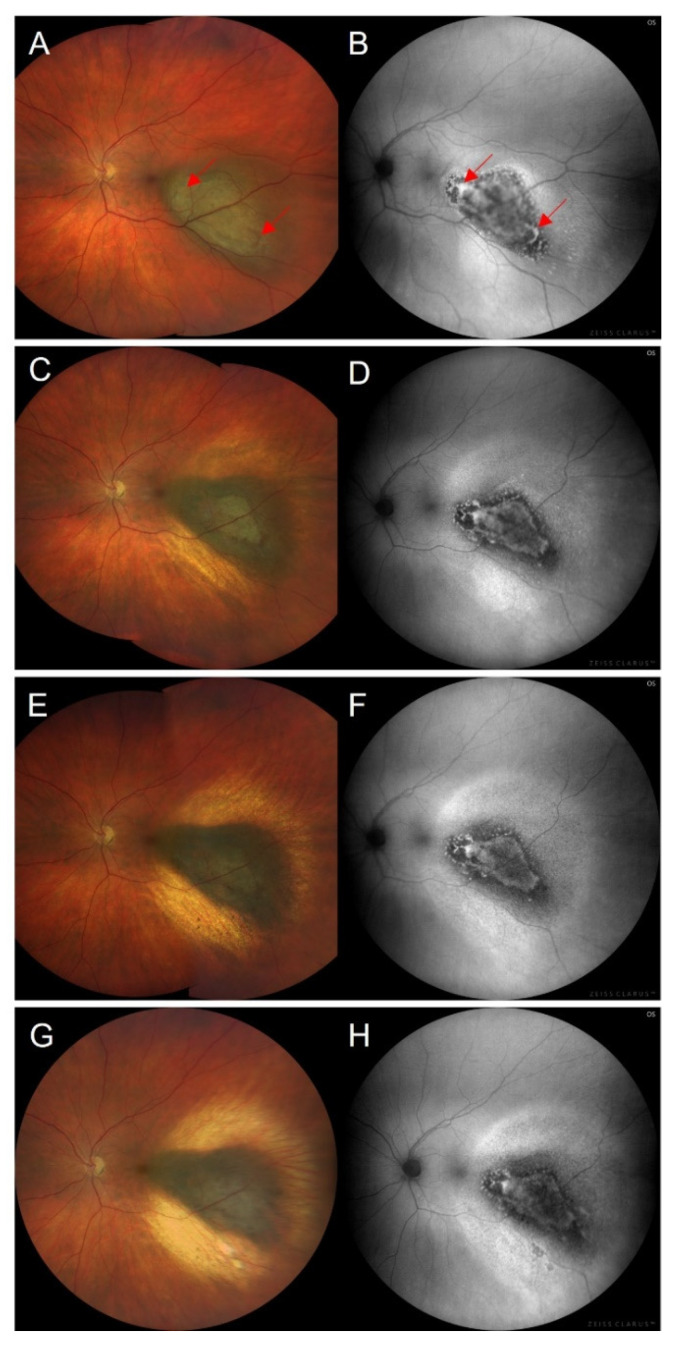
(**A**,**C**,**E**,**G**) Fundus photographs and (**B**,**D**,**F**,**H**) fundus autofluorescence images (illumination with 500–585 nm, FAF-Green) of a posterior choroidal melanoma (**A**,**B**) before and (**C**–**H**) after ruthenium-106 brachytherapy (CCA plaque, Eckert & Ziegler BEBIG, Berlin, Germany; scleral dosis 1600 Gy). (**C**,**D**) 3 months; (**E**,**F**) 9 months; (**G**,**H**) 18 months after brachytherapy. (**A**) The fundus photograph of the treatment-naive melanoma shows a pigmented lesion with overlying orange pigment (exemplified red arrows). (**C**,**E**,**G**) After brachytherapy, regression of the melanoma and surrounding chorioretinal scarring occurs. (**D**,**F**,**H**) Fundus autofluorescence imaging after therapy shows gradual fading of the increased autofluorescence signal of orange pigment and areas of progressive atrophy of the retinal pigment epithelium overlying the irradiated tumour. The surrounding irradiation field is characterised by increased fundus autofluorescence with a granular appearance. Fundus photographs and autofluorescence images were taken using the ZEISS CLARUS 700 (Carl Zeiss Meditec AG, Jena, Germany).

Clinical assessment of local tumour control post-treatment is mainly based on ultrasonography for measurements of the tumours’ horizontal and vertical dimensions in combination with fundus photography and OCT. FAF imaging following different treatment options for choroidal melanoma has not yet been addressed intensely. There are few reports on FAF imaging after plaque brachytherapy (Figure 4) and/or transpupillary thermotherapy, or after proton beam radiation therapy.

In a retrospective study, Gündüz et al. included two eyes treated with plaque radiotherapy and four eyes after combined plaque radiotherapy and transpupillary thermotherapy [87]. After a mean follow-up of 5 months (range 2–9 months), choroidal melanomas presented with increased FAF due to orange pigment and hyperpigmentation (HRA, Heidelberg Engineering, Dossenheim, Germany).

Similar results were reported by Amselem et al. following plaque radiotherapy in two patients and combined plaque radiotherapy and transpupillary thermotherapy in six patients with a mean follow-up of 4 months (range 2–9 months) [88]. The authors confirm the usefulness of FAF imaging to detect orange pigment and an increased FAF intensity after therapy. In addition, the published figures show extensive FAF changes, with areas of decreased FAF besides the postulated increased FAF. Due to the combination of therapies in these studies, the FAF effects are not assignable to a single therapy. Increased FAF following treatment was also mentioned by Cennamo et al. [79].

The regression patterns of choroidal melanoma after palladium-103 plaque brachytherapy have been described in a retrospective case series of 170 consecutive patients by Maheshwari and Finger, including a decrease in tumour thickness, an increase in tumour pigmentation, the diminution of tumour-related vascularity, resorption of subretinal fluid, and increasing internal reflectivity on ultrasonography post-therapy [89]. In addition, the presence of orange pigment was evaluated by means of FAF imaging pre- and post-therapy with a mean follow-up of 71 months (range 12–201 months). Interestingly, the last follow-up revealed an increasing number of eyes with a loss of orange pigment compared to the initial findings, whereas within the first 3 years after plaque brachytherapy, the presence of orange pigment was increased, which the authors named a crescendo–decrescendo fluctuation in the presence of orange pigment. Five to ten years after brachytherapy, the authors found an almost complete disappearance of orange pigment. In contrast, a solitary local recurrence was associated with the presence of new orange pigment. Therefore, newly appearing orange pigment may be considered as a sign of local recurrence.

**Figure 5:**
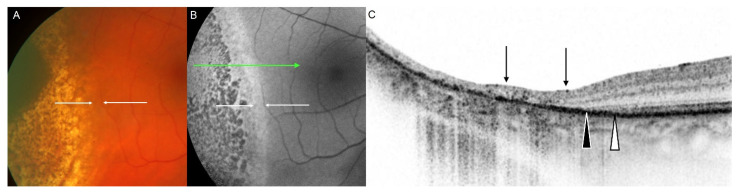
(**A**) Fundus photograph and (**B**) fundus autofluorescence image 6 months after ruthenium-106 brachytherapy (CCB plaque, Bebig GmbH, Berlin, Germany; scleral dose 650 Gy) of a choroidal melanoma with 4 mm tumour height before therapy in a 58-year-old female patient. (**A**) Visible changes on the fundus photograph encompass a smaller area, (**B**) compared to alterations seen on the fundus autofluorescence image. (**C**) The corresponding OCT scan shows loss of the ellipsoid zone (white arrowhead) and loss of the external limiting membrane (black arrowhead) that appear overlying an intact retinal pigment epithelial cell layer, and even outside visible changes on the fundus photograph and the fundus autofluorescence image. The area of temporal retinal atrophy is accompanied by changes at the level of the retinal pigment epithelium (e.g., RPE migration, RPE clumping, RPE atrophy). The fundus photograph was taken using FF 450^plus^ (Carl Zeiss Meditec AG, Jena, Germany); fundus autofluorescence and OCT: SPECTRALIS HRA+OCT, Heidelberg Engineering, Heidelberg, Germany). Reproduced from Bindewald-Wittich et al. [91] with permission from S. Karger AG, Basel.

**Figure 6:**
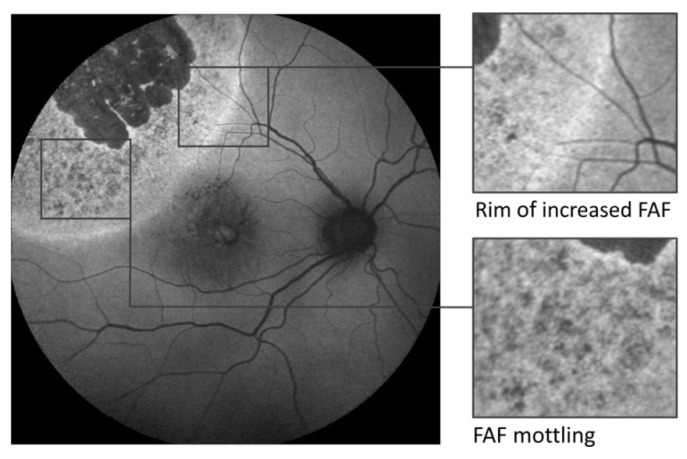
Central fundus autofluorescence image (excitation wavelength 488 nm) taken 45 months after ruthenium-106 brachytherapy (CCA plaque, Bebig GmbH, Berlin Germany; scleral dose 460 Gy) of a choroidal melanoma in a 52-year-old male. Areas with atrophy of the retinal pigment epithelium overlying the irradiated melanoma apex appear dark, whereas the farther irradiation field shows an inhomogeneous increased fundus autofluorescence signal with small, speckled areas of decreased autofluorescence (mottling). Furthermore, a rim of considerably increased autofluorescence clearly delineates the irradiation effect on retinal layers that contribute to the autofluorescence signal. The fundus autofluorescence image was taken using SPECTRALIS HRA+OCT, Heidelberg Engineering, Heidelberg, Germany. Reproduced from Bindewald-Wittich et al. [91] with permission from S. Karger AG, Basel.

Other reports underline the visualization of irradiation effects after plaque brachytherapy beyond ophthalmoscopically visible changes using FAF imaging [90,91]. In a retrospective study, both ruthenium-106 (*n* = 19) and iodine-125 (*n* = 2) plaque brachytherapy were found to induce a well-demarcated, increased FAF signal around the tumour in 69% of the eyes, detected by Optomap P200Tx (Optos, Dunfermline, UK) [90]. Another retrospective case series (*n* = 31) concretised the treatment effects of ruthenium-106 brachytherapy using confocal scanning laser FAF imaging and their correlation with optical coherence tomography (SPECTRALIS HRA + OCT, Heidelberg Engineering, Heidelberg, Germany) and fundus photography [91]. First, in 90.3% of the eyes, a rim of increased FAF demarcated the irradiation field of FAF and exceeded funduscopically visible changes (Figure 5). It was associated with a corresponding loss of the ellipsoid zone and the external limiting membrane on optical coherence tomography. Secondly, in 93.5% of the eyes, increased FAF with a speckled decreased FAF (FAF mottling) occurred within the irradiation field around the choroidal melanoma, due to photoreceptor cell loss (window defect) in combination with a thinning of the neurosensory retina and RPE degeneration, such as clumping, migration, and atrophy (Figure 6). Both FAF characteristics may be useful for the early detection of treatment effects and help to visualize accurate positioning of the used plaque. However, the occurrence of descriptive FAF changes may not be directly indicative for the applied irradiation dose. To date, the prognostic relevance of these FAF changes regarding long-term tumour control remains unclear. FAF characteristics that are relevant for local tumour control with respect to regression and signs of recurrence still need to be defined and analysed in prospective studies. Similarly, further studies are needed to determine the time of occurrence of early and late FAF changes following brachytherapy.

There is one report on ultra-wide-field imaging of choroidal melanoma before and after proton beam radiation therapy using green laser autofluorescence imaging (excitation wavelength 532 nm, detection of emission between 570 and 780 nm) [92]. In total, 97% of the choroidal melanomas showed a mixed autofluorescence intensity pre-therapy and 3% a reduced FAF signal. There was no significant change following proton beam radiation therapy. The authors explain that proton beam radiation therapy damages tumour cells and does not induce relevant RPE changes. Hence, direct treatment effects are not expected to change the FAF signal after proton beam radiation therapy.

## 4. Limitations

When performing FAF imaging in the presence of choroidal tumours, the following limitations need to be considered. At the time of initial diagnosis, most choroidal melanomas may still underlie the RPE, and the choroidal melanoma does not exhibit significant autofluorescence [59,60]. Hence, FAF imaging cannot image the tumour itself but can help to visualize secondary overlying RPE and retinal changes. Absorbing molecules within the optical path anterior to the tumour and a limited light penetration depth may impair the FAF imaging of deeper layers. Furthermore, the imaging of elevated lesions is challenging. Out-of-focus regions may not be imaged properly, especially using confocal imaging devices, because light originating from outside the confocal plane is suppressed [76]. Rather, prominent lesions may result in artefactual changes [65].

In general, media opacities (including cataract, opacified posterior lens capsule, asteroid hyalosis, vitreous debris, and vitreous haemorrhage) confound the FAF signal and can result in dramatically reduced image quality with impaired informative value. Adequate camera adjustment is crucial. Mean FAF images of confocal scanning laser ophthalmoscopes may be more susceptible to motion artifacts compared to single-flash images using a fundus camera. Most devices require pupil dilation, and short-wavelength excitation light—even if maximal retinal irradiation is well below the published exposure limits [36]—may be uncomfortable for the patient.

Although size measurements of lesions are possible, changes of the FAF intensity are evaluated in a descriptive manner and characterise relative deviations from the normal surrounding FAF signal. The nomenclature is partially non-uniform. Whereas some authors use the terms hyper-, iso- and hypoautofluorescent, others describe changes of the FAF signal as increased, normal, or decreased FAF compared to the normal FAF signal. The latter differentiate consciously between the nomenclature of FAF imaging (increased/decreased FAF) and fluorescence angiography (hyperfluorescent/hypofluorescent). However, both nomenclatures reflect the relative description of FAF intensities, and the interpretation of FAF images, especially the establishment of classifications, needs training and experience [93]. Uniform nomenclature and classification of FAF changes overlying choroidal melanoma pre- and post-treatment are desirable and need to be established. Furthermore, however, none of these studies used quantitative FAF to quantitatively analyse FAF intensities. Quantitative FAF imaging has been introduced to measure short-wavelength FAF intensities [94,95]. It is based on the normalisation of fundus grey levels to a fluorescent reference in the optical pathway. However, due to the sophisticated requirements, quantitative FAF imaging is not routinely applied today. Another novel imaging technology is fluorescence lifetime imaging ophthalmoscopy (FLIO), which captures metabolic and structural changes based on the measurement of lifetimes of intrinsic retinal autofluorescence [96,97]. To the best of our knowledge, there is no current report on fluorescence lifetimes in patients with choroidal melanoma. Therefore, a potential benefit of fluorescence lifetime imaging in eyes with choroidal melanoma still needs to be assessed in ongoing research. Finally, the herein cited studies on FAF imaging of choroidal melanomas differ in applied imaging devices and in cohort sizes, with some authors reporting rather small numbers of patients. Regarding the different applied excitation and emission spectra and technical differences, FAF images from different devices may not be completely equivalent. Further studies assessing the reliable comparability of FAF from different devices in the context of choroidal melanoma are still lacking. There is a need for prospective quantitative research on FAF imaging in the presence of choroidal melanoma.

## 5. Conclusions

FAF imaging is an important component of clinical multimodal imaging to detect, diagnose, and monitor choroidal melanoma. Using the autofluorescent properties of the ocular fundus, it gives additional information over and above conventional imaging modalities and is helpful to identify retinal and RPE changes associated with choroidal melanoma. In particular, the metabolic health status of the RPE and the occurrence of orange pigment overlying the tumour’s surface can be readily visualized by means of FAF imaging. As orange pigment is a well-established risk factor for malignant transformation, the detection of orange pigment using FAF has a relevant impact on clinical decision making. Larger tumours may be reliably diagnosed as malignant. Therefore, multimodal imaging, including FAF imaging, is particularly helpful to accurately diagnose and monitor small choroidal melanomas. Additionally, FAF imaging is adjuvant to visualize the biological effects of radiotherapy on the choroidal tumour and adjacent healthy tissue. Future clinical research is needed to further assess the benefit of FAF imaging of choroidal melanoma before and after treatment. Continuing prospective and long-term studies should aim to establish reliable diagnostic characteristics and uniform nomenclature and classification of FAF changes associated with untreated and treated choroidal melanoma.

## Figures and Tables

**Table 1:** Study characteristics. Technical devices, study design, number of eyes, and major findings using fundus autofluorescence imaging of choroidal nevi and melanomas.

Study	Technical Device	Excitation Wavelength	Detection of Emission	Number of Eyes	Design	Major FAF Findings
Shields et al. [66]	Fundus camera *(ZEISS)*	580 nm	>695 nm	4 small melanomas (2 pigmented, 2 nonpigmented)	Four case reports	Tumour itself isoautofluorescent. Orange pigment with discrete, bright increased autofluorescence.
Gündüz et al. [76]	cSLO *(HRA, Heidelberg Engineering*)	488 nm	>500 nm	23 (12 melanomas, 11 nevi)	Retrospective chart review	~90% of tumours showed at least 1 focus of increased FAF. Orange pigment in 13 eyes was correlated with increased FAF: completely in 8 tumours (61.5%), partially in 3 tumours (23.1%), not in 2 tumours (15.4%).
Gündüz et al. [80]	cSLO, *(HRA, Heidelberg Engineering*)	488 nm	>500 nm	30 (19 melanomas, 11 nevi)	Retrospective chart review	Patchy FAF pattern: areas of increased FAF between areas of normal FAF. Diffuse FAF pattern: increased FAF with indistinct borders. Nevi (*n* = 11) presented with patchy FAF pattern. Melanomas presented with patchy (*n* = 6) and diffuse (*n* = 13) FAF pattern. Orange pigment was present in 15 eyes.
Samuelsson et al. [81]	Fundus camera *(TRC-50DX,* *Topcon)*	535–585	605–715 nm	49 (19 nevi, 10 IML, 10 melanomas, 10 other choroidal tumours)	Retrospective study	Melanomas revealed a patchy (*n* = 6) and a diffuse (*n* = 4) FAF pattern. Significant differences were observed between choroidal nevi, IML, choroidal melanomas, and other choroidal tumours regarding FAF findings.
Lavinsky et al. [65]	cSLO *(HRA2, Heidelberg Engineering)*	488 nm	>500 nm	20 (5 melanomas, 15 nevi)	Retrospective observational study/consecutive case series	Nevi were associated with normal FAF. Melanomas were associated with confluent to plaque-like increased FAF due to orange pigment.
Cennamo et al. [79]	cSLO *(SPECTRALIS HRA+OCT, Heidelberg Engineering)*	488 nm	>500 nm	165 (65 melanomas, 100 nevi)	Letter to the editor	Nevi: normal (*n* = 40) or decreased (*n* = 60) FAF. Melanomas: plaque-like increased FAF and surrounding decreased FAF. Orange pigment: brightly increased FAF with adjacent dark areas with decreased FAF.
Shields et al. [82]	Fundus camera *(Carl Zeiss Meditec)*	580 nm	>695 nm	64 nevi	Consecutive case series	Decreased FAF in 56%, normal FAF in 19%, and increased FAF in 25%.
Shields et al. [83]	Fundus camera *(Carl Zeiss Meditec)*	580 nm	>695 nm	51 melanomas	Non-comparative case series	Decreased FAF in 39%, normal FAF in 6%, and increased FAF in 55%.
Reznicek et al. [84]	SLO *(Panoramic 200Tx, Optos)*	532 nm	540–800 nm	139 (101 melanomas, 38 nevi)	Consecutive case series	Mean FAF intensity of choroidal melanomas was significantly lower than the mean FAF intensity of choroidal nevi.

FAF: fundus autofluorescence. cSLO: confocal scanning laser ophthalmoscope; SLO: scanning laser ophthalmoscope; IML: indeterminate choroidal melanocytic lesions.
